# Effect of steam explosion on nutritional composition and antioxidative activities of okra seed and its application in gluten‐free cookies

**DOI:** 10.1002/fsn3.1739

**Published:** 2020-06-30

**Authors:** Lei Hu, Jiamin Guo, Xiwei Zhu, Rui Liu, Tao Wu, Wenjie Sui, Min Zhang

**Affiliations:** ^1^ State Key Laboratory of Food Nutrition and Safety Tianjin University of Science & Technology Tianjin China; ^2^ Jing Hong Yuan Modern Agricultural Technology Co., Ltd. Hengshui Hebei Province China; ^3^ Engineering Research Center of Food Biotechnology Ministry of Education Tianjin China; ^4^ Tianjin Agricultural University Tianjin China

**Keywords:** antioxidative activities, gluten‐free cookies, in vitro enzymatic digestion, okra seed, steam explosion

## Abstract

Health‐conscious consumers are increasingly interested in gluten‐free (GF) foods. Raw okra seed (ROS) flour and steam‐exploded okra seed (SEOS) flour were explored for developing GF cookies with high nutritional values and in vitro enzymatic digestion. Results indicated that the steam explosion exhibited significant effects on enhancing the release of dietary fibers and lipids in okra seed flour at moderate explosion pressure. Although steam explosion caused the loss of flavonoid compounds, moderate high explosion pressure enhanced the release of total phenolics ranged from 294.57 to 619.07 mg GAE/100 g DM with significantly improved DPPH^•^ radical scavenging activity (from 18.78% to 67.34%) and ferric reducing antioxidant power (from 13.37% to 149.04%). The rapidly digestible starch (RDS) content in GF cookies decreased with increasing steam explosion severity, whereas slowly digestible starch (SDS) and resistant starch (RS) contents significantly increased from 36.91% to 40.92% and from 2.50% to 9.06%, respectively. Steam explosion is an effective technique for enhancing the release of nutrients like dietary fiber and total phenolics, and okra seed flour, especially SEOS flour, can be alternative choices to provide food functional materials for developing various GF food products.

## Graphical abstract

Raw okra seed flour and steam‐exploded okra seed (SEOS) flour were explored for developing gluten‐free (GF) cookies with high nutritional values and in vitro enzymatic digestion. Steam explosion is an effective technique for enhancing the release of nutrients like dietary fiber and total phenolics, and okra seed flour, especially SEOS flour, can be alternative choices to provide food functional materials for developing various GF food products.

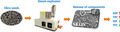

## 1 INTRODUCTION

Recently, health‐conscious consumers are increasingly interested in gluten‐free (GF) cereal‐based foods, because of the increasing signs and symptoms of gluten intolerance, such as fatigue, bone or joint pain, and weight fluctuation, in addition to celiac disease in the gastrointestinal tract. The food industry has made positive responses to improve the formulas of GF foods and remove gluten from the diet for people who are gluten intolerant (O'Shea, Doran, Auty, Arendt, & Gallagher, 2013). The GF cereals, including rice, corn, and sorghum flours, have been used for developing food products, like GF rice cookies, for celiac patients (Giuberti et al., 2018; Jnawali, Kumar, & Tanwar, 2016). However, following a GF diet generally signifies removing gluten from the diet for a life‐long modification and may lead to a lower intake of important components for health benefits (Foschia, Beraldo, & Peressini, 2016). Several studies have suggested that GF foods presented lower resistant starch and dietary fiber contents than their gluten‐containing equivalents (Foschia et al., 2016; Giuberti et al., 2018). Therefore, it is important to enhance the overall nutritional, processing adaptability, and sensory acceptability of GF food products.

Nowadays, seed flours have been utilized for improving the nutritional values of GF foods, especially digestive resistance (Arribas et al., 2017; Awolu, Sudha, & Manohar, 2018; Giuberti et al., 2018). For instance, Goyat, Passi, Suri, and Dutta (2018) developed chia and quinoa seed flour‐substituted cookies, which enrich in phenolics, flavonoids, and antioxidants with acceptable sensory properties. Besides, Simons and Hall III (2018) reported that cookies that are made with flours containing 40% raw pinto beans were acceptable at the same level as cookies made with pretreated pinto beans, thereby reducing the cost of inputs. Okra (*Abelmoschus esculentus* [Linn.] Moench) is originally cultivated in Africa and now grown in different tropical and warm subtropical regions of Europe, Africa, the Middle East, India, and Southeast Asia. Previous studies have shown that okra seeds showed high contents of dietary fiber, protein, polyphenols, and flavonoids and thus possessed antioxidative, antidiabetic, and antidepressant benefits (Savello, Martin, & Hill, 1980; Tongjaroenbuangam et al., 2011; Xia et al., 2018). According to Petropoulos et al. (2017), okra seeds could be potential rich sources of dietary fiber, protein, and bioactive compounds that benefit to antioxidative and antimicrobial properties. However, the nutritional value of okra seeds has not been fully understood, and the application of okra seeds in GF foods has not been explored.

Some indications showed that steam explosion is a new and effective technique for improving the nutritional values of food byproducts and their processing foods (Sui, Xie, Liu, Wu, & Zhang, 2018). Fernández‐Bolaños, Felizón, Brenes, Guillén, and Heredia (1998) have demonstrated that phenolic compounds characteristic of olive stone (hydroxytyrosol) increased with increasing steaming temperature and time. Zhang, Yang, Zhao, Hua, and Zhang (2014) reported that steam explosion significantly improved protein extraction yield from soybean meal, and emulsifying properties of steam explosion‐treated protein were enhanced owing to the changes protein structures. Song et al. (2014) employed a high‐density steam explosion to extract flavonoids from pine needles and achieved 2.54‐fold higher extraction yield as that of the untreated sample. Xu and Chang (2008) indicated that the pressure steaming rather than steam explosion resulted in significant increases in total phenolic content (TPC), total flavonoid content (TFC), condensed tannin content and in vitro antioxidative activities in yellow soybeans but a decrease in black soybean, which might be due to long‐time thermal effects on black soybean. Our previous studies also showed that the steam explosion decreased insoluble dietary fiber (IDF) content but increased soluble dietary fiber (SDF) content, thereby benefiting to the extraction of water‐soluble bioactive compounds (Sui et al., 2018). Steam explosion is a typical combined technique of hydrothermal reactions and mechanically tearing effects, which demonstrated that steam explosion might have certain benefits, including increasing nutritional values, changing protein and fiber structures, and reducing particle sizes and improving sensory characteristics (Guo et al., 2015). To our knowledge, there is little information concerning the effect of steam explosion on legumes and seeds, especially for okra seeds, and the incorporation of okra seed flour in GF formulations for the development of functional GF food products.

This study aimed to evaluate the nutritional composition and antioxidative activities of steam‐exploded okra seed (SEOS) as a dietary fiber ingredient in GF cookies. The in vitro enzymatic digestion, textural and sensory characteristics of GF cookies in the presence of steam explosion‐treated okra seed flour were also studied as compared with those of pure GF cookies without the addition of okra seed flour and those of GF cookies with the addition of nonsteam explosion okra seed flour. This study will provide knowledge about the employment of steam explosion for improving the nutritional values of legumes and seeds, which is useful for food raw material producers and manufacturers to develop new formulations of GF food products.

## 2 MATERIALS AND METHODS

### 2.1 Materials

The commercially available dried okra seeds (*A. esculentus* [Linn.] Moench) were purchased from the local market in Bozhou City (Anhui Province, China), and the commercial rice flour from Indica rice (*Oryza sativa* L.) was purchased from Jianhao Food Co., Ltd. (Fujian Province, China). The proximate composition of rice flour is as follows: moisture 6.02% ± 0.08%, protein 12.60% ± 0.37%, lipid 0.01% ± 0.00%, ash 0.13% ± 0.01%, and total starch 74.97% ± 0.47%. All other materials were of food‐grade and were obtained from the local market in Tianjin City (China). Unless mentioned otherwise, all the chemicals used were of analytical grade and were purchased from National Pharmaceutical Group Corporation (Beijing City, China). All the enzyme assay kits were purchased from Megazyme Ltd. (Dublin, Ireland).

### 2.2 Steam explosion process

Prior to steam explosion treatment, okra seeds were soaked with distilled water at a ratio of okra seeds to distilled water 1:1 (w/v). The steam explosion process was performed on a QBS‐200B type steam explosion device (Hebei Gentle ICSE EnvironTech Co. Ltd.) with the reactor volume of 5,000 ml. The device consists of a reactor chamber, a receiver, and a steam generator. 500 g rehydrated okra seeds were loaded into the reactor chamber, treated with saturated steam at steam pressures of 1.0, 1.5, and 2.0 MPa for 5 min, respectively and suddenly exploded and decompressed into the receiver within 0.1 s. The SEOSs samples were collected and dried in the oven at 50°C for 4 hr. The raw okra seed (ROS) and SEOS samples were then ground to pass through a 250 μm diameter mesh screen, and the ROS flour and SEOS flour products were stored at 4°C for further use.

### 2.3 Chemical composition analysis of okra seed flour

#### 2.3.1 Chemical composition

The moisture content was determined according to AOAC 934.01 loss on drying method (AOAC, 2005). The crude protein, crude lipid, and ash content were determined by the Kjeldahl method (AOAC 976.05), the ether extraction method (AOAC 954.02), and the ashing method (AOAC 942.05), respectively (AOAC, 2000). The total starch content was analyzed by the amyloglucosidase/α‐amylase method with Megazyme assay kit K‐TSTA 06/17, according to AOAC 996.11 (AOAC, 2000).

#### 2.3.2 Dietary fiber composition

The total dietary fiber (TDF), SDF, and IDF contents were determined using Megazyme assay kit K‐RINTDF 10/15 and following manufacturer’s assay procedure.

#### 2.3.3 Fatty acid composition

The fatty acid composition was analyzed by preparing fatty acid methyl esters (FAME) according to the direct method reported by O'Fallon, Busboom, Nelson, and Gaskins (2007). The FAME composition was determined on a GCMS‐QP2010 gas chromatograph‐mass spectrometer (GC‐MS) (Shimadzu Corporation) equipped with a BR‐SWax capillary column (30 m × 0.32 mm × 0.5 μm) for FAME. The injection volume was 1 μl with a split ratio of 5:1 at the injection temperature of 250°C and the N_2_ flow of 2 ml/min. The oven temperature was programmed as follows: 60°C for 5 min, then 10°C/min until 245°C for 20 min. The MS was performed with electron ionization at the temperature of 220°C, the scan speed of 1,000 u/s, acquisition mass range of 35–500 u, scan interval of 0.5 s, and solvent delay of 4 min. The peak was identified using a Supelco 37 component FAME mix (Sigma‐Aldrich). The percentages of fatty acids were calculated with GC‐MS peak areas by area normalization method according to AOAC 963.22 method (AOAC, 2000).

### 2.4 Bioactive properties of okra seed flour

#### 2.4.1 Total phenolic and flavonoid content

One gram of ROS and SEOS flour was mixed with 20 ml distilled water and then treated by high‐speed shearing on an IKA‐T25 homogenizer (UltraTurrax IKA) at 8,000 rpm for 5 min. Extracts were centrifuged at 2,673 *g* for 10 min at 4°C. The resulting supernatant was then dissolved with distilled water to a constant volume of 25 ml and regarded as okra seed extracts for further total phenolic, TFC, and antioxidative activity determination.

Total phenolic content and TFC of ROS and SEOS flour were determined according to Sarker and Oba (2018) with the okra seed extracts diluted 10 times and 5 times, respectively. TPC was expressed as mg gallic acid equivalents per 100 g dry matter (mg GAE/100 g DM), and TFC was expressed as mg rutin equivalents per 100 g dry matter (mg RE/100g DM).

#### 2.4.2 In vitro antioxidative activities

In vitro antioxidative activities of ROS and SEOS were evaluated in terms of DPPH^•^ and $O_{2}^{\cdot -}$ radical scavenging activity (RSA), and ferric reducing antioxidant power (FRAP). DPPH^•^ RSA was determined following the method of Sharma, Saxena, and Riar (2016) with the okra seed extracts diluted 50 times. The $O_{2}^{\cdot -}$ RSA was determined as described by Zhu et al. (2018) with the okra seed extracts diluted 10 times. FRAP assay of okra seed extracts was conducted using a modified method of Contreras‐Calderón, Calderón‐Jaimes, Guerra‐Hernández, and García‐Villanova (2011) without dilution.

### 2.5 Microstructure imaging of okra seed flour

ROS and SEOS flour samples were frozen in a –80°C freezer for 4 hr. Afterward, samples were mounted on aluminum stubs and sputter‐coated by gold in a Hummer XP vacuum evaporator (Anatech). The microstructure of ROS and SEOS flour samples were visualized on a Hitachi SU‐1510 scanning electron microscopy at a beam accelerating voltage of 15 kV with magnifications of ×500 and ×1,000.

### 2.6 Physical properties of GF cookies

#### 2.6.1 GF cookies formulation

The base ingredient with different addition ratios of okra seed flour to rice flour (0:100, 2:98, 4:96, and 6:94) was prepared based on rice flour + okra seed flour 100 g, butter 30 g, egg 55 g, baking powder 2 g, salt 1 g, and distilled water 6 g. For all formulas, no sugar was added to limit the amount of glycemic carbohydrates (Giuberti et al., 2018). The mixture was mixed with an HM740 mixer (Hauswirt) to form a dough. The dough was sheeted to 4 mm thickness and then cut into cookies pieces with an approximately 42 mm in diameter circular mold. Cookies pieces were baked using a Xingdu YXD‐30C business oven (Shandong, China) at a temperature of 180°C for 18 min and then cooled and stored in separate plastic bags at room temperature until further analysis. The GF cookie without okra seed flour was regarded as pure GF cookies, while ROS flour‐fortified GF cookies at different addition ratios of okra seed flour to rice flour (2:98, 4:96, and 6:94) were presented as ROS‐GF‐2, ROS‐GF‐4, and ROS‐GF‐6 cookies (Figure S1); SEOS flour‐fortified GF cookies at different SE treatment conditions (1.0, 1.5, and 2.0 MPa) and different addition ratios of okra seed flour to rice flour (2:98, 4:96, and 6:94) were presented as SEOS1.0‐GF‐2, SEOS1.5‐GF‐4, and SEOS2.0‐GF‐6 cookies, respectively.

#### 2.6.2 Spread ratio

Weights of all GF cookies were determined by ±0.0001 g accuracy analytical balance. Thickness, diameter, and spread ratio were measured according to Mancebo and Gmezauthor (2016).

#### 2.6.3 Rheological measurement

All GF cookies were subjected to the rheological measurement on a Haake MARS III rheometer (Thermo‐Scientific) equipped with a 20 mm in diameter parallel plate geometry gapped by 1 mm. The changes in storage modulus (*G*′), loss modulus (*G″*), and loss factor (tan*δ*) in the frequency range of 0.1–100 rad/s were determined by a preliminary strain sweep test at 1 Hz and 25°C (Li, Liu, Wu, Wang, & Zhang, 2016).

#### 2.6.4 Color

The color determination of GF cookies was performed on an NR10QC Chroma Meter (Shenzhen 3NH Technology Co.Ltd.) and the L* (lightness), a* (redness‐greenness) and b* (yellowness‐blueness) values were obtained separately.

#### 2.6.5 Textural properties

Textural analyses, including hardness and brittleness, were performed on a TA. XT. Plus texture analyzer (Stable Micro System) equipped with an HDP/3PB probe, using 3 mm/s of pretest speed, 3 mms/s of test speed, and 10 mm/s post‐test speed in automatic trigger mode at 5 g of trigger force.

### 2.7 In vitro enzymatic digestion of GF cookies

All GF cookies samples were ground to pass through an 80‐mesh screen to simulate the chewing process. In vitro enzymatic digestion of all GF cookies was determined according to the method of Guo, Yu, Copeland, Wang, and Wang (2018) with slight modification. Briefly, 100 mg flour sample was mixed with 4 ml NaAc buffer (pH 5.2) at a temperature of 37°C for 25 min prior to the addition of 1 ml mixed enzyme solution. The mixed enzyme solution was prepared as follows: 1.3 g *α*‐amylase (15 U/mg, Sigma‐Aldrich) was dissolved in 11.85 ml distilled water with stirring at 37°C for 10 min and centrifuged at 2,673 *g* for 10 min. 8 ml supernatant was then mixed with 0.1 ml amyloglucosidase (3,150 U/ml, Megazyme Resistant Starch assay kits K‐RSTAR 02/17). The amount of released glucose was measured at 0, 20, and 120 min with a D‐glucose assay kit (GOPOD, Megazyme Resistant Starch assay kits K‐RSTAR 02/17). The rapidlydigestible starch (RDS), slowly digestible starch (SDS), and resistant starch (RS) contents were calculated following the below equations.

$$\text{RDS}\;\left(\%\right)=[\left(G_{20}-\text{FG}\right)/\text{TS}]\times 0.9\times 100$$

$$\text{SDS}\;\left(\%\right)=\left[(G_{120}-G_{20})/\text{TS}\right]\times 0.9\times 100$$

$$\text{RS}\;\left(\%\right)=[\left(\text{TS}-G_{120}\right)/\text{TS}]\times 0.9\times 100$$

where FG and TS represented the free glucose, and total glucose amount in the sample, respectively; G_20_ and G_120_ were the released glucose amount after 20 min and 120 min enzymatic hydrolysis, respectively.

### 2.8 Sensory evaluation of GF cookies

Sensory properties of GF cookies were evaluated by 10 panelists (five males and five females, 30–35 years old) from the College of Food Science and Engineering, using a 9‐point hedonic scale for four attributes (color, texture, flavor, and taste). 9‐point represented “like extremely,” whereas 1‐point represented “dislike extremely.” Finally, the overall performance was calculated as a summed score of the four indicators and used to evaluate the sensory quality of GF cookies.

### 2.9 Statistical analysis

Unless otherwise specified, experiments were performed in triplicate with results expressed as mean ± standard deviation. Statistical analyses were conducted using one‐way analysis of variance (ANOVA) and Duncan's multiple range tests with the help of SPSS software (version 17, SPSS Inc.). Differences were considered significant at *p* < .05.

## 3 RESULTS AND DISCUSSION

### 3.1 Chemical properties of okra seed flour

#### 3.1.1 Proximate composition

The proximate compositions of ROS and SEOS flour were shown in Table 1. It presented that ROS flour had significantly (*p* < .05) higher content of protein (38.00%) and lipid (14.14%) as compared to rice four (12.60% and 0.01%), as well as a higher percentage of TDF (37.67%), including SDF (5.10%) and IDF (32.56%). The moisture, protein, and ash content of the studied ROS flour were within the range of different genotypes of okra seeds reported in previous literature (Petropoulos et al., 2017), while higher total dietary fiber (37.67%) and lower lipid content (14.14%) were observed. However, it was in agreement with the result of (Savello et al., 1980), in which the lipid and carbohydrate contents were 13.58% and 60.04% for ground okra meal, and 25.57% and 35.56% for sifted okra seed meal. The inconsistent results might be attributed to different genotypes, storage period, and/or grinding‐sieving process parameters, which were important for nutritional value.

**Table 1:** Proximal composition, total phenolic (TPC), total flavonoid content (TFC), and in vitro antioxidative activities of ROS and SEOS flour

Sample	ROS	SEOS1.0	SEOS1.5	SEOS2.0
Moisture (%)	7.70 ± 0.06^a^	7.74 ± 0.09^a^	6.45 ± 0.11^b^	5.97 ± 0.05^c^
Protein (%)	38.00 ± 1.01^a^	17.55 ± 0.44^d^	19.62 ± 0.65^c^	21.26 ± 0.15^b^
Lipid (%)	14.14 ± 0.18^b^	11.58 ± 0.57^c^	15.07 ± 0.84^b^	16.35 ± 0.19^c^
Ash (%)	5.53 ± 0.22^a^	4.54 ± 0.22^d^	5.44 ± 0.19^c^	5.47 ± 0.28^b^
Total starch (%)	0.16 ± 0.01^b^	0.14 ± 0.00^c^	0.16 ± 0.01^b^	0.17 ± 0.01^a^
TDF (%)	37.67 ± 0.41^d^	51.20 ± 0.19^a^	40.48 ± 0.53^b^	39.16 ± 0.25^c^
SDF (%)	5.10 ± 0.05^d^	5.90 ± 0.71^c^	6.50 ± 0.73^a^	6.05 ± 0.48^b^
IDF (%)	32.56 ± 0.36^d^	45.30 ± 0.60^a^	33.98 ± 1.22^b^	33.12 ± 0.33^c^
TPC (mg GAE/100 g DM)	522.47 ± 1.09^c^	294.57 ± 1.09^d^	528.16 ± 2.89^b^	619.07 ± 4.76^a^
TFC (mg RE/100 g DM)	42.22 ± 0.11^a^	32.61 ± 0.23^b^	26.30 ± 0.34^b^	22.74 ± 0.29^d^
DPPH^•^ RSA (%)	18.78 ± 0.36^d^	35.50 ± 0.69^c^	46.19 ± 0.95^b^	67.34 ± 0.17^a^
$O_{2}^{\cdot ‐}$ RSA (%)	6.31 ± 0.06^b^	2.34 ± 0.33^d^	6.28 ± 0.18^c^	6.35 ± 0.06^a^
FRAP (mmol/L)	13.37 ± 0.92^d^	24.93 ± 0.66^c^	83.97 ± 2.44^b^	149.04 ± 2.01^a^

Note

Mean values followed by a different superscript in the same row are significantly different (*p* < .05).

Abbreviations: DM, dry matter; FRAP, ferric reducing antioxidant power; GAE, gallic acid equivalents; IDF, insoluble dietary fiber; ROS, raw okra seed; RE, rutin equivalents; RSA, radical scavenging activity; SDF, soluble dietary fiber; SEOS, steam‐exploded okra seed; TDF, total dietary fiber.

#### 3.1.2 Dietary fiber composition

The pretreatment of okra seeds could also affect the proximate composition of okra seed flour. Table 1 shows the proximate composition of SEOS flour samples under different steam explosion conditions (1.0, 1.5, and 2.0 MPa of explosion pressure). It can be observed that the SEOS flour had higher TDF content, including SDF and IDF contents, but lower protein and moisture contents than those of ROS flour. TDF and IDF contents increased to the maximum values of 51.20% and 45.30%, respectively, at explosion pressure of 1.0 MPa, while SDF content reached the maximum value of 6.50% at explosion pressure of 1.5 MPa; however, all of them began to decrease with the further increase of explosion pressure. It is widely acknowledged that the steam explosion treatment can trigger various hydrothermal reactions during the cooking stage, such as starch gelatinization, thermal protein denaturation, and cell walls destruction (Sui et al., 2018). Gong, Huang, and Zhang (2012) also reported an increase in water‐soluble carbohydrates under relatively moderate explosion conditions, owing to the hydrolysis of insoluble hemicelluloses and celluloses into soluble components, including oligosaccharides, monosaccharides, and degradation products. In contrast, a reduction of soluble carbohydrates was observed under more drastic explosion conditions to form carboxylic acids or soluble polymers by a series of secondary reactions. On the contrary, protein and lipid contents firstly decreased to minimum values of 17.55% and 11.58%, respectively, at explosion pressure of 1.0 MPa and then increased with raising explosion pressure. Proteins and lipids bind to other components in foods, such as starch and non‐starch polysaccharides, forming complexes that are resistant to the extraction procedures and leading to the reduction of protein and lipid contents at explosion pressure of 1.0 MPa (Arribas et al., 2017). Since the depolymerization degree of celluloses, hemicelluloses, and lignin enhanced with the increasing of explosion pressure, resulting in excessive leakage of components in cells or cell walls, the protein and lipid contents had an upward trend under explosion pressure conditions of above 1.0 MPa.

#### 3.1.3 Fatty acid composition

The fatty acid compositions of ROS and SEOS flour were shown in Table 2. The result of fatty acid compositions was similar to that reported in the study of (Petropoulos et al., 2017). The ratio of polyunsaturated to saturated fatty acids (PUFA/SFA) ranged from 1.14 to 1.21, implying high nutritional value (Guil, Torija, Giménez, & Rodríguez, 2009). While the amount of *n*‐3 fatty acids was dramatically lower than that of *n*‐6 fatty acids, which is typical for most vegetable oils. After steam explosion treatment, a slight decrease in total SFA and total monounsaturated fatty acids (MUFA) contents of okra flour was observed at each explosion pressure value (1.0, 1.5, and 2.0 MPa). Correspondingly, SEOS flour samples showed higher PUFA value respect to ROS flour counterparts, especially for *n*‐3 and *n*‐6 PUFA, which are essential and must be supplied by the food diet (Giuberti et al., 2018). It was worth noting that conversion reactions among fatty acids occurred during the steam explosion treatment of SEOS flour. C18:0 and C18:1 *n*‐9 *cis* fatty acids were mainly reduced during the steam explosion. At meanwhile, C20:0, C18:1 *Tran* and C18:3 *n*‐3 have observed in the SEOS flour sample obtained at 1.0 MPa (SEOS1.0) and 2.0 MPa (SEOS2.0), while C22:1 *cis* were formed in the SEOS flour sample obtained at 1.5 MPa (SEOS1.5). Studies have reported that a high intake of trans fatty acids contributed to the risk of coronary heart disease (Oomen et al., 2001), the production of trans fatty acids during the process of the steam explosion should be avoided as possible.

**Table 2:** Fatty acid composition analysis of ROS and SEOS flour

Sample	ROS	SEOS1.0	SEOS1.5	SEOS2.0
Total SFA	35.49	33.82	35.11	34.43
C14:0	0.38	0.52	0.47	0.46
C16:0	31.56	30.44	31.42	31.02
C17:0	0	0.19	0.27	0.18
C18:0	3.55	2.48	2.95	2.52
C20:0	0	0.19	0	0.25
Total MUFA	24.06	23.78	23.96	23.77
C16:1	1.81	1.95	1.27	1.94
C18:1 n‐9 *cis*	22.25	21.57	21.38	21.46
C18:1 *trans*	0	0.26	0	0.37
C22:1 *cis*	0	0	1.31	0
Total PUFA	40.45	42.40	40.93	41.80
C18:2 n‐6 *cis*	40.45	42.05	40.93	41.44
C18:3 n‐3	0	0.35	0	0.36

Note:

SEOS flour samples obtained at different SE treatment conditions (1.0, 1.5, and 2.0 MPa) were presented as SEOS1.0, SEOS1.5, and SEOS2.0, respectively.

Abbreviations: MUFA, monounsaturated fatty acids; PUFA, polyunsaturated fatty acids; ROS, raw okra seed; SEOS, steam‐exploded okra seed; SFA, saturated fatty acids.

Taking the above results into account, SEOS flour showed significantly (*p* < .05) higher dietary fiber content, but possessed lower protein content than that of ROS flour. Besides, the SEOS flour treated under explosion pressure above 1.0 MPa had higher lipid content than that of ROS flour. The results indicated that steam explosion treatment was conducive to the release of dietary fiber and lipids at moderate explosion pressure, accompanied by the formation of large cavities and intercellular spaces, which effectively help extraction of the soluble components in okra seeds. Besides, in comparison with ROS, SEOS1.5 showed the closest fatty acid composition.

### 3.2 Bioactive properties of okra seed flour

#### 3.2.1 Total phenolic and flavonoid content

Total phenolic contents of ROS and SEOS flour (Table 1) ranged from 294.57 to 619.07 mg GAE/100 g DM. As shown in Table 1, SEOS1.0 had significantly lower (*p* < .05) TPC than that of ROS, implying that the exposure of ROS to high‐pressure saturated steam led to the loss of phenolic structures and activities. However, TPC released tended to be higher as steam explosion treatment severity conditions increased to 1.5 and 2.0 MPa in materials pre‐impregnated with water. As suggested in the report by Conde et al. (2009), part of the phenolic compounds were probably released as substituents of the oligosaccharides solubilized. Thus, TPC obtained free from sugars increased at increasing steam explosion severity. Among the SEOS flour, TPC values were significantly (*p* < .05) higher in the SEOS1.5 and SEOS2.0 in comparison with ROS, followed by SEOS1.0, suggesting that steam explosion could produce a considerable increase of TPC in okra seed flour and the extent depended upon the steam explosion conditions.

Total flavonoid contents of ROS and SEOS flour (Table 1) were significantly (*p* < .05) different, ranging from 22.74 to 42.22 mg RE/100 g DM. ROS showed the highest TFC value of 42.22 mg RE/100 g DM in comparison with SEOS, and the TFC value of okra seed flour decreased with the explosion pressure increased, suggesting that high‐pressure saturated steam caused the damage of flavonoid compounds during the steam explosion, which is a typically hydrothermal treatment (Sui & Chen, 2014).

#### 3.2.2 In vitro antioxidative activities

In vitro antioxidative activities, including DPPH^•^ RSA, $O_{2}^{\cdot ‐}$ RSA, and FRAP, of ROS and SEOS flour were evaluated (Table 1) and ranged from 18.78% to 67.34% (DPPH^•^ RSA), 2.34% to 6.35% ($O_{2}^{\cdot ‐}$ RSA), and 13.37 to 149.04 mmol/L (FRAP). It could be seen that increasing the steam explosion severity led to an increase in DPPH^•^ RSA and FRAP. SEOS2.0 showed the highest antioxidant efficiency in DPPH^•^ RSA, followed by SEOS1.5, SEOS1.0, and ROS, with significant differences (*p* < .05) observed. The order of FRAP for ROS, SEOS1.0, SEOS1.5, and SEOS2.0 resembled that of DPPH^•^ assay, while the changing trend of $O_{2}^{\cdot ‐}$ RSA was not consistent with that of DPPH^•^ RSA and FRAP, showing the order of SEOS2.0 > SEOS1.5 > ROS > SEOS1.0.

### 3.3 Correlations between bioactive and chemical properties

Correlations among IDF, SDF, TPC, TFC, and antioxidative activities (DPPH^•^ RSA, $O_{2}^{\cdot ‐}$ RSA, and FRAP) were conducted to describe the effects of different explosion pressures (0, 1.0, 1.5 and 2.0 MPa) on antioxidative activities of okra seed flour. As shown in Table 3, TPC had a negatively linear correlation with IDF content (*R* ^2^ = −.945), confirming that celluloses, hemicelluloses, and lignins were degraded into oligosaccharides, monosaccharides, and other degradation products at relatively higher explosion pressure of 1.5 and 2.0 MPa. It should be noted that TPC assay is not specific for polyphenols, as any reducing agent, such as lignin derivatives (Vinardell, Ugartondo, & Mitjans, 2008), and Maillard reaction derivatives formed (Amorati & Valgimigli, 2015), may react with Folin reagent (Savello et al., 1980). Therefore, along with the increase of steam explosion severity, the increasing amounts of hydrothermal degradation products might also be counted into TPC, indicating the reverse correlation between IDF and TPC (*R* ^2^ = −.872). Moreover, TFC exhibited a reverse correlation with SDF content in certain pressure ranges (0−1.5 MPa) of the steam explosion, which confirmed that relatively moderate steam explosion caused an increase of water‐soluble carbohydrates but notably damage of flavonoid compounds.

**Table 3:** The correlations between TPC, TFC, DPPH^•^ RSA, $O_{2}^{\cdot ‐}$ RSA (%), and FRAP in okra seed flour samples at four different explosion conditions (0, 1.0, 1.5, and 2.0 MPa)

	IDF (%)	TPC (mg GAE/100 g DM)	TFC (mg QE/100 g DM)	DPPH^•^ RSA (%)	$O_{2}^{\cdot ‐}$ RSA (%)	FRAP (mmol/L)
SDF (%)	.105	.067	−.872^#^	.699^#^	−.018**	.599
IDF (%)		−.945^#^	.062	−.167	−.996** ^,^ ^#^	−.421
TPC (mg GAE/100 g DM)			−.338	.468	.951* ^,^ ^#^	.685^#^
TFC (mg QE/100 g DM)				−.960*	−.132	−.893^#^
DPPH• RSA (%)					.219	.964*
$O_{2}^{\cdot ‐}$ RSA (%)						.466

Abbreviations: DM, dry matter; FRAP, ferric reducing antioxidant power; GAE, gallic acid equivalents; IDF, insoluble dietary fiber; IDF, insoluble dietary fiber; RSA, radical scavenging activity; SDF, soluble dietary fiber; TFC, total flavonoid content; TPC, total phenolic content.

** Correlation is significant at a level of .01 (two‐tailed).

* Correlation is significant at a level of .05 (two ‐tailed).

^#^ Coefficients of correlation are above .6 (*R* ^2^ > .6).

A highly significant correlation (*R* ^2^ = −.960) was observed between DPPH^•^ RSA and TFC. Despite the decrease of TFC along with the explosion pressure, DPPH^•^ RSA exhibited a significantly upward trend and showed certain correlations with SDP content (*R* ^2^ = .699) and TPC (*R* ^2^ = .468), respectively. This result indicated that SEOS1.0 might be subjected to severe destruction of the cell wall and, to a certain extent, modification of IDF into SDF, leading to the enhanced release of other antioxidant compounds, along with the increase of SDF, for enhancing DPPH^•^ RSA. The higher DPPH^•^ RSA of SEOS1.5 and SEOS2.0 might be mainly attributed to hydrothermal reaction products formed during the steam explosion. In particular, part of lignins was degraded and converted into pigment derivatives, and insoluble phenolic compounds were released from hemicellulose, causing the production of free phenolic compounds (Castro et al., 2008; Gong et al., 2012).

Similarly, Garrote, Cruz, Domínguez, and Parajó (2008) reported that the release of phenolics occurred at higher severity than that of sugars in barley husks, suggesting that moderate high steam explosion severity is beneficial to the formation of soluble phenolic compounds. Furthermore, $O_{2}^{\cdot ‐}$ RSA showed highly significant correlations with IDF (*R* ^2^ = −.996) and significantly correlations with TPC (*R* ^2^ = .951), which indicated that the phenolics were main contributors to the antioxidant activities of SEOS flour for $O_{2}^{\cdot ‐}$ RSA. In addition, FRAP showed a similar trend with that of DPPH^•^ RSA (*R* ^2^ = .964), possessing negatively correlation with TFC and positively correlation with TPC. TFC had higher correlation coefficient with $O_{2}^{\cdot ‐}$ RSA than DPPH^•^ RSA and FRAP, which suggested that some nonphenolics, such as reducing sugars formed during steam explosion, were also contributed to the antioxidant activities of okra seed flour for DPPH^•^ RSA and FRAP. This trend was different from the result of (Gong et al., 2012), who found that TPC and FRAP were highly positively correlated (*r* = .918). The difference could be due to the different resources studied and the different steam explosion parameters used. Therefore, although steam explosion caused the loss of flavonoid compounds, moderate high explosion pressure enhanced the release and production of soluble reducing sugars and total phenolics with good antioxidative activities.

### 3.4 Microstructure of okra seed flour

**Figure 1:**
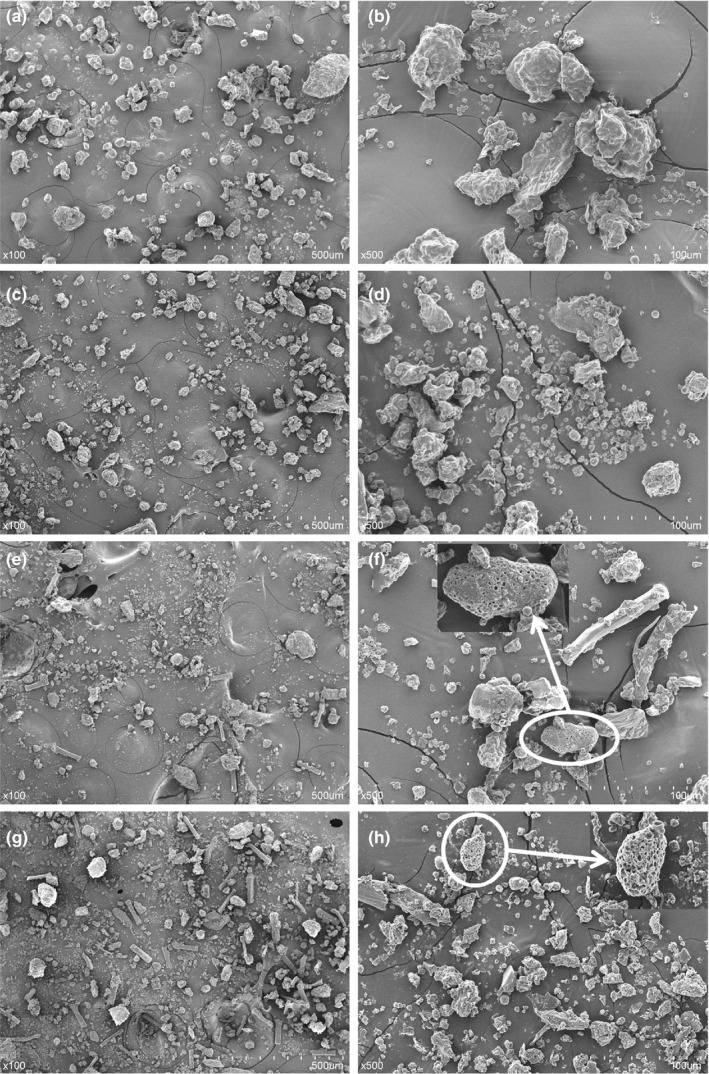
*SEM* images of ROS (a, b) and SEOS flour treated at different explosion pressures of 1.0 MPa (c, d), 1.5 MPa (e, f), and 2.0 MPa (g, h) with magnifications of ×100 an ×500, respectively. Parts of SEOS flour samples in the circle were imaged with a magnification of ×1,000

*SEM* micrographs of SEOS flour were shown in Figure 1, compared with ROS flour as a control sample. As shown in Figure 1, particles of ROS and SEOS flour samples were <250 μm since samples were passed through a 250 μm screen. The particle surfaces of ROS flour sample were complete and relatively smooth (Figure 1a,b). After the steam explosion at 1.0 MPa, more small particles were observed with some faults and cavities (Figure 1c,d). With increasing the severity of the steam explosion, the particle size of SEOS flour samples became further finer, and the microstructure of SEOS flour samples was more porous at explosion pressure of 1.5 and 2.0 MPa, in comparison with that of SEOS1.0 (Figure 1e–h). The insects of Figure 1f and h clearly showed the formation of obvious cavities and voids due to the breakage and destruction of cell walls, as well as the dissolution of internal soluble components during the steam explosion process (Chen & Chen, 2011). This is a good explanation for the increase in SDF, TPC, and antioxidative activities of SEOS flour. Besides, rod‐like and fibrous structures were found in the SEOS1.5 sample and were extensively formed in the SEOS2.0 sample, as shown in Figure 1e and g. According to Abraham et al. (2011), the removal of cementing materials like hemicellulose and lignin could cause the increase of crystallinity of the fiber. Under severe explosion pressures of 1.5 and 2.0 MPa, the remaining crystalline particles were isolated (Abraham et al., 2011), and the amorphous cellulose probably became more crystalline (Carrasco et al., 1994).

### 3.5 Physical properties of GF cookies

#### 3.5.1 Spread ratio and rheological properties

**Figure 2:**
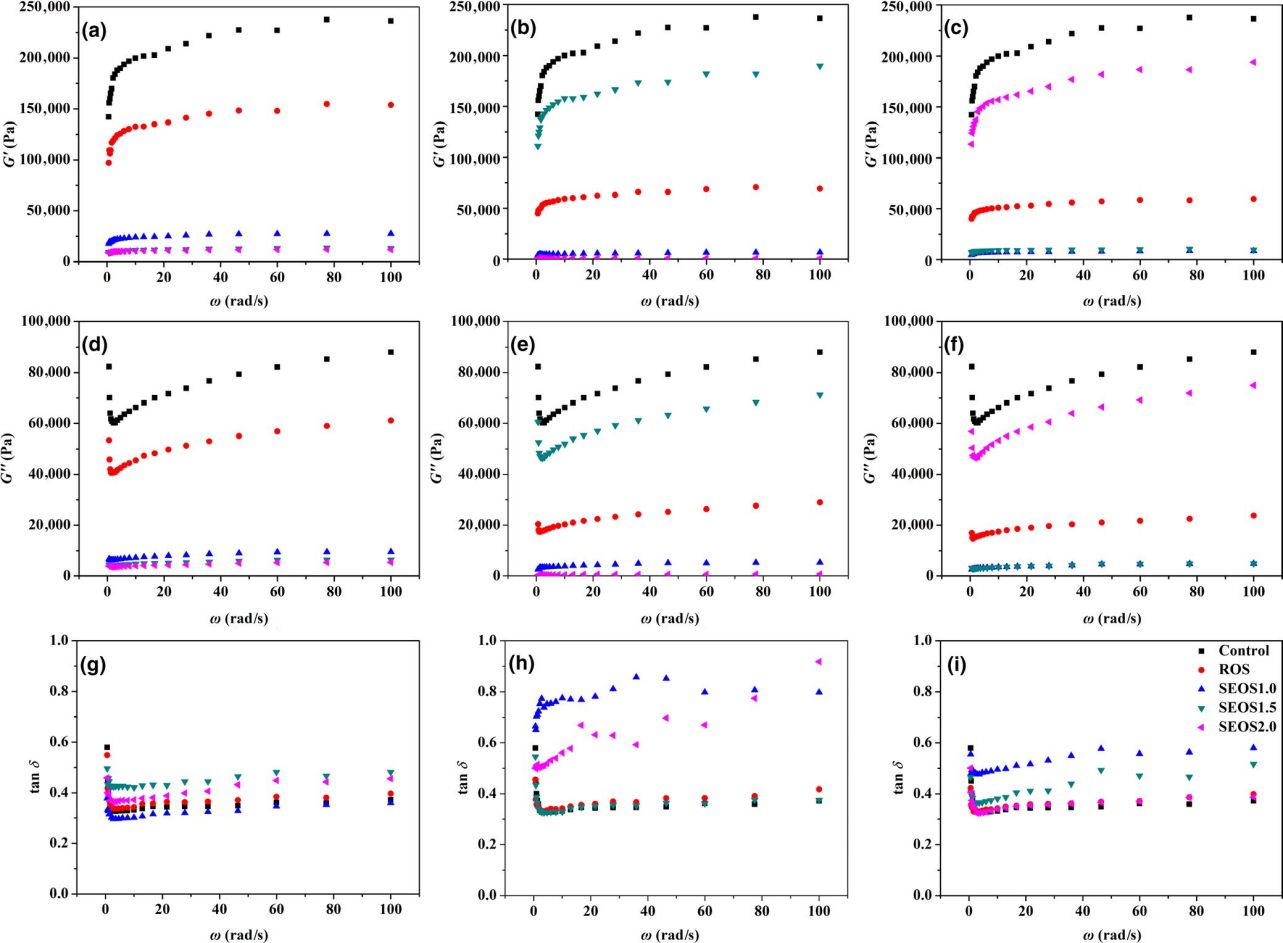
Storage modulus, *G*′, loss modulus, *G*″, and loss factor, tan*δ* of GF dough systems with the addition of 2% (a, d, g), 4% (b, e, h), and 6% (c, f, i) of okra seed flour

The potential of ROS and SEOS flour in GF cookies formulation was evaluated by determining the physical and textural properties of GF cookies, as well as rheological properties of dough (Table 4 and Figure 2). As shown in Table 4, combining ROS and SEOS flour in GF cookies had slightly higher spread ratio values than that of pure GF cookies. It suggested that ROS or SEOS‐fortified GF cookies possessed a higher spread ratio, which is beneficial to cookies quality. When the addition amount of okra seed flour was 2% and 6%, the spread ratio of ROS and SEOS‐fortified GF cookies showed no significant difference with that of pure GF cookies; meanwhile, SEOS1.0‐GF‐4 and SEOS1.5‐GF‐4 had significantly higher spread ratios of 10.57 and 10.55 than that of ROS‐GF‐4 and pure GF cookies. In general, the cookies with high viscosity showed relatively low spread ratio, as indicated by the rheological results (Figure 2) that ROS and SEOS‐fortified GF cookies owned lower *G*″ values than that of pure GF cookies. For all amounts of okra seed flour (2%, 4%, and 6%), both *G′* and *G*″ values of ROS and SEOS‐fortified dough decreased with the increase of explosion steam severity, except for the addition of 4% of SEOS1.5 and 6% of SEOS2.0. According to Figure 2, storage modulus *G′* was higher than loss modulus *G″* (tan*δ* < 1), indicating the elastic‐like behavior of GF cookie dough. However, tan*δ* values of GF cookie dough notably increased by adding SEOS flour, yield a GF cookie dough, which became less elastic (O'Shea et al., 2013). In particular, the addition of 2% SEOS1.0, 4% SEOS1.5, and 6% SEOS2.0 had little influence on the elastic properties of GF cookie dough. It could be attributed to the interaction effects of various components in okra seed flour and rice flour, such as phenolics, flavonoids, SDF, IDF, sugars, and protein (Liu, Shi, Song, Wu, & Zhang, 2018).

**Table 4:** Physical and textural properties of GF cookies with different addition amounts of ROS and SEOS flour

Cookies sample (g)	Diameter (mm)	Thickness (mm)	Spread ratio	L*	a*	b*	Hardness
Pure GF	42.88 ± 0.48^ab^	4.35 ± 0.14^a^	9.86 ± 0.36^bc^	70.46 ± 0.24^a^	5.93 ± 0.18^f^	43.36 ± 0.25^a^	716.22 ± 8.28^a^
ROS‐GF‐2	42.78 ± 0.41^abc^	4.35 ± 0.17^a^	9.84 ± 0.46^bc^	64.22 ± 0.79^b^	9.25 ± 0.12^a^	42.18 ± 0.70^b^	351.71 ± 7.04^g^
ROS‐GF‐4	42.62 ± 0.22^abc^	4.31 ± 0.12^a^	9.90 ± 0.27^bc^	60.34 ± 0.97^d^	8.88 ± 0.21^b^	35.99 ± 0.62^d^	442.91 ± 9.26^d^
ROS‐GF‐6	42.68 ± 0.25^abc^	4.22 ± 0.20^ab^	10.14 ± 0.61^abc^	59.11 ± 0.62^e^	9.36 ± 0.10^a^	34.73 ± 0.47^e^	450.51 ± 13.31^d^
SEOS1.0‐GF‐2	42.48 ± 0.50^abc^	4.14 ± 0.03^ab^	10.27 ± 0.11^abc^	61.73 ± 0.96^c^	8.51 ± 0.09^c^	38.29 ± 0.59^c^	336.20 ± 12.03^g^
SEOS1.0‐GF‐4	42.30 ± 0.45^bc^	4.00 ± 0.13^b^	10.57 ± 0.20^a^	59.14 ± 1.14^de^	8.49 ± 0.24^c^	33.13 ± 0.30^f^	371.76 ± 6.51^f^
SEOS1.0‐GF‐6	42.38 ± 0.40^bc^	4.15 ± 0.07^ab^	10.20 ± 0.13^abc^	55.68 ± 0.99^f^	8.54 ± 0.18^c^	30.27 ± 0.64^h^	448.09 ± 12.35^d^
SEOS1.5‐GF‐2	42.72 ± 0.47^abc^	4.14 ± 0.09^ab^	10.33 ± 0.26^ab^	59.12 ± 0.83^de^	7.93 ± 0.17^e^	34.50 ± 0.59^e^	422.16 ± 17.84^e^
SEOS1.5‐GF‐4	42.36 ± 0.41^bc^	4.02 ± 0.19^b^	10.55 ± 0.54^a^	53.12 ± 0.92^g^	8.23 ± 0.06^d^	29.64 ± 0.24^j^	461.01 ± 5.76^d^
SEOS1.5‐GF‐6	43.04 ± 0.28^a^	4.15 ± 0.19^ab^	10.40 ± 0.55^ab^	52.07 ± 0.65^gh^	8.18 ± 0.13^d^	27.57 ± 0.22^j^	518.90 ± 7.60^c^
SEOS2.0‐GF‐2	42.22 ± 0.33^c^	4.64 ± 0.19^a^	9.74 ± 0.43^c^	55.67 ± 0.97^f^	8.23 ± 0.20^d^	32.44 ± 0.46^g^	458.09 ± 1.51^d^
SEOS2.0‐GF‐4	42.80 ± 0.44^abc^	4.16 ± 0.15^ab^	10.30 ± 0.35^abc^	51.67 ± 0.54^h^	8.47 ± 0.14^c^	29.30 ± 0.44^i^	502.97 ± 7.27^c^
SEOS2.0‐GF‐6	42.80 ± 0.38^abc^	4.09 ± 0.19^b^	10.43 ± 0.51^ab^	47.04 ± 0.99^i^	8.52 ± 0.09^c^	27.05 ± 0.29^j^	589.72 ± 12.69^b^

Note:

Mean values followed by a different superscript in the same column are significantly different (*p* < .05).

Abbreviations: GF, gluten‐free; ROS, raw okra seed; SEOS, steam‐exploded okra seed.

#### 3.5.2 Color

In terms of the cookie color (Table 4), L* and b* values decreased, whereas a* values increased in ROS and SEOS‐fortified GF cookies with respect to pure GF cookies, indicating a browning color of cookies with the addition of ROS and SEOS. The natural pigmentation of ROS flours resulted in the browning of ROS‐fortified GF cookies, as a function of the addition amounts. Besides, Maillard reactions during baking also played an important role in browning color formation, which was correlated with the protein content, sugar content, and other reaction subtracts. As shown in Table 4, SEOS‐fortified GF cookies presented significantly lower L*, a*, and b* values, in comparison with ROS‐fortified GF cookies, and the L*and b* values decreased along with both the addition amount of okra flour and the severity of the steam explosion. It indicated that the steam explosion caused more browning subtracts in SEOS flour, like pigments and reducing sugars, as indicated by the results of in vitro antioxidative activities. Therefore, the incorporation of ROS and SEOS in GF cookies could lead to the reduction of lightness and browning changes due to the higher Maillard reaction subtracts formed during the steam explosion.

#### 3.5.3 Textural properties

The textural properties of cookies were in accordance with the dimensional and rheological properties (Table 4). ROS and SEOS‐fortified cookie dough with weaker and less elastic rheological property yielded weaker cookies with more extensible dimensions. Furthermore, the replacement of rice flour with increasing levels of okra seed flour increased the hardness of GF cookies. However, the hardness of GF cookies firstly decreased from ROS to SEOS1.0 and then increased with increasing the severity of steam explosion. With the increase of steam explosion pressure, the changing trend of hardness was consistent with that of protein content. According to Giuberti et al. (2018), higher protein contents could contribute to a harder structure as from the strong interactions between proteins and other components in GF cookies. Besides the protein content, the presence of fibers also affected hardness. Higher fiber content, as indicated by rod‐like and fibrous structures in Figure 1, can probably contribute to a more compact dough structure.

### 3.6 In vitro enzymatic digestion of GF cookies

The digestion behaviors of GF cookies with or without ROS and SEOS flour were investigated. The RDS, SDS, and RS contents of pure GF, ROS, and SEOS‐fortified GF cookies with different addition amounts (0%, 2%, 4%, and 6%) of okra seed flour are shown in Table 5. In comparison with pure GF cookie, ROS and SEOS‐fortified GF cookies presented lower RDS content but higher SDS and RS contents, due to low starch content and poor digestibility based on the inherent composition in okra seed, as well as interaction effects between starch and other food components in GF cookies during the cooking process. As a result, the RDS content significantly decreased with increasing the addition of ROS and SEOS flour, while SDS and RS contents accordingly increased. For each addition amount of okra seed flour, the RDS content was reduced as the severity of steam explosion enhanced, whereas SDS and RS increased. Since the enzymatic hydrolysis ability of *α*‐amylase was closely related to the aggregation arrangement of starch molecules, the results indicated that starch and other components in okra seed like proteins and phenolics formed a structure more resistant to digestion (Jiranuntakul, Puttanlek, Rungsardthong, Punchaarnon, & Uttapap, 2011; Liu et al., 2017), as indicated by the result of in vitro antioxidative activities. It has reported that SDS and RS have nutritional functions for human bodies. For example, SDS usually provides a prolonged release of glucose, and RS consumption improves gut health, adiposity, and insulin resistance (Keenan et al., 2015).

**Table 5:** In vitro enzymatic digestion and sensory evaluation of GF cookies with different addition amounts of ROS and SEOS flour

Cookies sample	RDS (%)	SDS (%)	RS (%)	Color	Texture	Flavor	Taste	Overall acceptability
Pure GF	50.59 ± 0.43^a^	36.91 ± 0.52^f^	2.50 ± 0.11^h^	7.20 ± 0.63^a^	5.90 ± 0.57^a^	6.40 ± 0.84^a^	6.10 ± 0.99^b^	6.20 ± 0.63^c^
ROS‐GF‐2	46.64 ± 0.22^b^	38.69 ± 0.20^e^	4.67 ± 0.33^f^	5.90 ± 0.99^bcd^	6.30 ± 0.95^a^	6.60 ± 0.97^a^	6.50 ± 0.97^ab^	6.30 ± 0.82^bc^
ROS‐GF‐4	45.00 ± 0.51^c^	39.49 ± 0.46^cde^	5.51 ± 0.05^e^	5.70 ± 1.06^cd^	6.50 ± 1.08^a^	6.50 ± 0.97^a^	6.70 ± 0.82^ab^	6.30 ± 0.67^bc^
ROS‐GF‐6	42.84 ± 0.21^d^	40.35 ± 0.43^ab^	6.81 ± 0.23^c^	5.60 ± 1.07^cd^	6.30 ± 1.16^a^	6.10 ± 0.74^a^	6.40 ± 1.07^ab^	6.40 ± 0.52^bc^
SEOS1.0‐GF‐2	46.45 ± 0.44^b^	39.38 ± 0.49^de^	4.17 ± 0.29^g^	5.70 ± 1.95^cd^	6.10 ± 0.99^a^	6.00 ± 1.15^a^	6.50 ± 0.97^ab^	6.60 ± 0.52^abc^
SEOS1.0‐GF‐4	44.98 ± 0.45^c^	39.24 ± 0.38^de^	5.78 ± 0.07^de^	5.20 ± 0.63^d^	6.20 ± 0.92^a^	5.90 ± 0.88^a^	6.60 ± 0.97^ab^	6.60 ± 0.70^abc^
SEOS1.0‐GF‐6	40.54 ± 0.18^e^	40.72 ± 0.41^a^	8.75 ± 0.33^a^	5.20 ± 1.03^d^	6.00 ± 1.05^a^	5.60 ± 0.97^a^	6.40 ± 0.84^ab^	6.70 ± 0.95^abc^
SEOS1.5‐GF‐2	44.98 ± 0.49^c^	39.04 ± 0.94^de^	5.98 ± 0.45^d^	5.30 ± 1.06^d^	6.20 ± 1.32^a^	5.80 ± 1.23^a^	6.40 ± 0.84^ab^	6.90 ± 0.74^abc^
SEOS1.5‐GF‐4	42.42 ± 0.36^d^	40.29 ± 0.52^abc^	7.29 ± 0.17^b^	5.40 ± 1.17^d^	5.90 ± 1.37^a^	6.40 ± 1.07^a^	6.80 ± 1.03^ab^	7.00 ± 0.82^abc^
SEOS1.5‐GF‐6	40.50 ± 0.56^e^	40.38 ± 0.44^ab^	9.12 ± 0.17^a^	5.20 ± 1.03^d^	6.20 ± 1.03^a^	6.10 ± 0.99^a^	6.40 ± 0.84^ab^	7.20 ± 0.92^a^
SEOS2.0‐GF‐2	44.67 ± 0.18^c^	40.49 ± 0.10^a^	4.84 ± 0.20^f^	6.50 ± 1.08^abc^	5.90 ± 0.99^a^	6.20 ± 1.32^a^	6.80 ± 1.03^ab^	7.10 ± 0.88^ab^
SEOS2.0‐GF‐4	42.76 ± 0.49^d^	39.62 ± 0.42^bcd^	7.62 ± 0.08^b^	6.70 ± 1.06^ab^	5.80 ± 1.23^a^	6.10 ± 0.99^a^	7.20 ± 1.14^a^	7.00 ± 0.82^abc^
SEOS2.0‐GF‐6	40.02 ± 0.27^e^	40.92 ± 0.21^a^	9.06 ± 0.07^a^	7.10 ± 1.10^a^	5.90 ± 1.20^a^	6.20 ± 1.14^a^	7.40 ± 0.84^a^	7.00 ± 0.67^abc^

Note:

Mean values followed by a different superscript in the same column are significantly different (*p* < .05).

Abbreviations: GF, gluten‐free; RDS, rapidly digestible starch; ROS, raw okra seed; RS, resistant starch; SDS, slowly digestible starch; SEOS, steam‐exploded okra seed.

### 3.7 Sensory evaluation of GF cookies

The sensory evaluation of GF cookies was conducted by a 9‐point hedonic scale for four attributes, including color, texture, flavor, and taste. The results of sensory evaluation are shown in Table 5. It could be seen from Table 5 that SEOS1.5‐GF‐6 had the highest overall acceptability among all GF cookies. The overall acceptability of SEOS1.5‐GF‐6 was significantly higher than that of pure and ROS‐fortified GF cookies, whereas all SEOS1.0, SEOS1.5, and SEOS2.0‐fortified GF cookies showed no significant difference in overall acceptability. Although ROS and SEOS‐fortified GF cookies had no significant differences in texture, flavor, and taste, the obvious browning color of SEOS flour contributed to the reduction in color perception. SEOS2.0‐GF‐6 presented a good taste but weak texture, probably owing to the higher sweetness from sugars formed during the steam explosion, which cause the cookie texture to harden.

## 4 CONCLUSION

Based on the above results, it can be concluded that the steam explosion exhibited significant effects on enhancing the release of dietary fibers and lipids in okra seed flour at moderate explosion pressure. Although the steam explosion resulted in the loss of flavonoids in okra seed flour, relatively high explosion pressure improved the production of soluble reducing sugars and total phenolics with strong antioxidative activities. The SEOS flour presented a more porous structure at higher explosion pressure with the formation of obvious cavities and voids. ROS and SEOS‐fortified cookie dough yield weaker cookies with more extensible dimensions and lower hardness. Furthermore, RDS content in GF cookies decreased with increasing the steam explosion severity, whereas SDS and RS accordingly increased. Combined with sensory evaluation results, SEOS1.5 flour showed relatively higher dietary fiber and lipid contents without transfatty acids, as well as moderately higher in vitro antioxidative activities and porous microstructure, which contributed to significantly higher SDS and RS content of SEOS1.5‐GF‐6 cookies, in comparison with those of pure and ROS‐fortified GF cookies. Therefore, the steam explosion is an effective technique for enhancing the release of nutritional components like dietary fiber and total phenolics in okra seeds, and okra seed flour, especially SEOS flour, can be alternatives to provide new food functional materials for developing various GF food products with a formulation adapted to the needs of gluten‐sensitive and health‐diet conscious consumers.

## CONFLICT OF INTEREST

The authors declare no conflicts of interest.

## Supporting information

Figure S1
