# Supplementary material for: Effect of steam explosion on nutritional composition and antioxidative activities of okra seed and its application in gluten‐free cookies
Source: Food Sci Nutr. 2020 Jun 30;8(8):4409–21. doi: 10.1002/fsn3.1739 (PMC7455944; doi:10.1002/fsn3.1739)
Supplement: Supplementary file 1 — Figure S1 [file FSN3-8-4409-s001.doc]

**Supporting Information**

**(A)
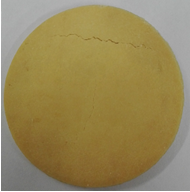
 (B)
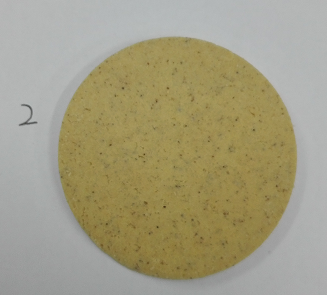
**

**(C)
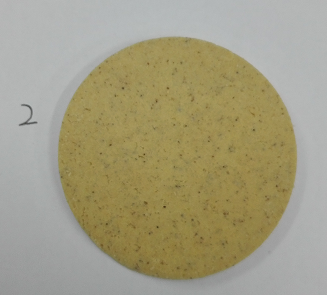
 (D)
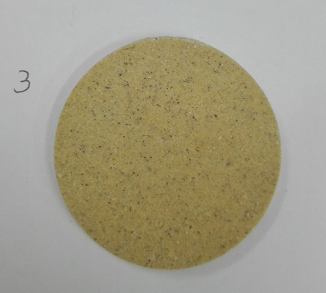
**

**(E)**
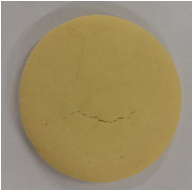
 **(F)**
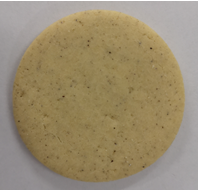


**(G)**
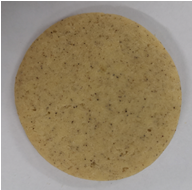
 **(H)**
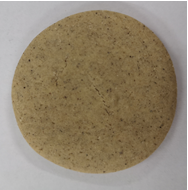


**Figure S1** Photographs of gluten-free cookies with 0% (A), 2% (B), 4% (C), 6% (D) of ROS flour and 0% (E), 2% (F), 4% (G), 6% (H) SEOS1.0 flour.
